# A national data linkage study to assess the extent, nature and outcomes of serious and organised crime prosecuted before the criminal courts in England and Wales

**DOI:** 10.23889/ijpds.v8i2.2204

**Published:** 2023-09-14

**Authors:** Tim McSweeney

**Affiliations:** 1 University of Hertfordshire, Hatfield, United Kingdom

## Objectives

The social and economic costs associated with serious and organised crime (SOC) are considerable: recently estimated by the National Audit Office to be £37 billion annually. Despite this, we know little about the extent and nature of SOC being prosecuted before the courts, or the outcomes associated with these cases.

## Method

Secondary analysis of over 12.6 million linked, individual-level, de-identified records from the Data First initiative relating to the criminal courts and prison system in England and Wales (E&W) over an eight-year period (2013-2020).

## Results

This unique study provides estimates for the rate and volumes of SOC appearing before the Crown Court in E&W and describes the characteristics of the defendants charged with these offences, and their associated outcomes. Using a comparative design, the study also assessed the severity and geographic distribution of offending associated with SOC; the extent to which prosecutions were discontinued, dismissed, or resulted in an acquittal (and the factors most predictive of this outcome); and the rate and frequency of reappearances before the criminal courts over time.

## Conclusion

The findings contribute towards enhancing government and law enforcement resilience in this area by developing a better understanding of SOC and the effectiveness of responses to it. This evidence informs key Ministry of Justice priorities linked to the effective and efficient delivery of justice and upholding the rule of law.

